# Prevalence of myocardial crypts in a cardiac magnetic resonance population - a large cohort study

**DOI:** 10.1186/1532-429X-15-S1-P95

**Published:** 2013-01-30

**Authors:** Tina Muhr, Nick Child, Eva Sammut, Darius Dabir, Eike Nagel, Valentina O Puntmann

**Affiliations:** 1 Cardiovascular Imaging, King's College London, London, UK

## Background

Myocardial crypts are discrete clefts or fissures in otherwise compacted myocardium of the left ventricle (LV) commonly located in basal inferior or inferoseptal segments. Few small studies reported higher prevalence of crypts in patients with hypertrophic cardiomyopathy (HCM) or their unexpressed relatives, and linked crypts to a marker of genotype positive phenotype negative carrier status. The exact clinical significance of crypts is unclear. We assessed the prevalence of myocardial crypts seen in consecutive subjects referred for cardiac magnetic resonance (CMR).

## Methods

We analyzed a cohort of consecutive subjects referred for clinical CMR during a period of 9 months (n=730, males: 443). Based on pre-test probability and CMR findings (low pretest probability and normal findings vs. high pretest probability and ischemic or non-ischemic cardiomyopathy), 3 groups were formed and examined for the presence of myocardial crypts. The non-ischemic cardiomyopathy group was further stratified into subgroups of identified etiology. Myocardial crypts were described according to their shape (i-, u- and v-form), depth (30-50%, 50-70%, >70% of myocardial diameter) and the segment location.

## Results

Overall, myocardial crypts were identified in 136 subjects (19%) with no gender difference (males: n=81, 18%; females: 55, 19%, p=0.78). Crypts were similarly proportioned in normal group (n=44,17%) and those with ischemic (n=37,22%) or non-ischemic cardiomyopathy (n=55,18%) (p=0.40). In the latter group, crypts were most common in subjects with HCM (n=19/62, 31%) again p - value against the other groups. In relatives to the patients with confirmed genetic cardiomyopathy only 11% crypts were found. Crypts most commonly presented by the u-shape (45%), followed by the v - (40%) and i - shape (15%). Sixty-percent of crypts were 30-50% deep, followed by 50-70% (35%) and 5 % > 70% myocardial diameter. Most of the myocardial crypts could be identified in the basal inferior segment (49%). In twenty percent, crypts were multiple, with no difference in representation in health or disease (p=0.22-0.89).

## Conclusions

In a large study cohort we demonstrate that myocardial crypts are similarly prevalent in subjects with and without cardiomyopathy. A subanalysis of non-ischaemic group reveals disproportionate representation with bias towards HCM. Contrasting previous reports, crypts were less common in relatives. Prospective studies are needed to confirm the clinical significance of myocardial crypts, if any.

## Funding

NIHR

**Figure 1:**
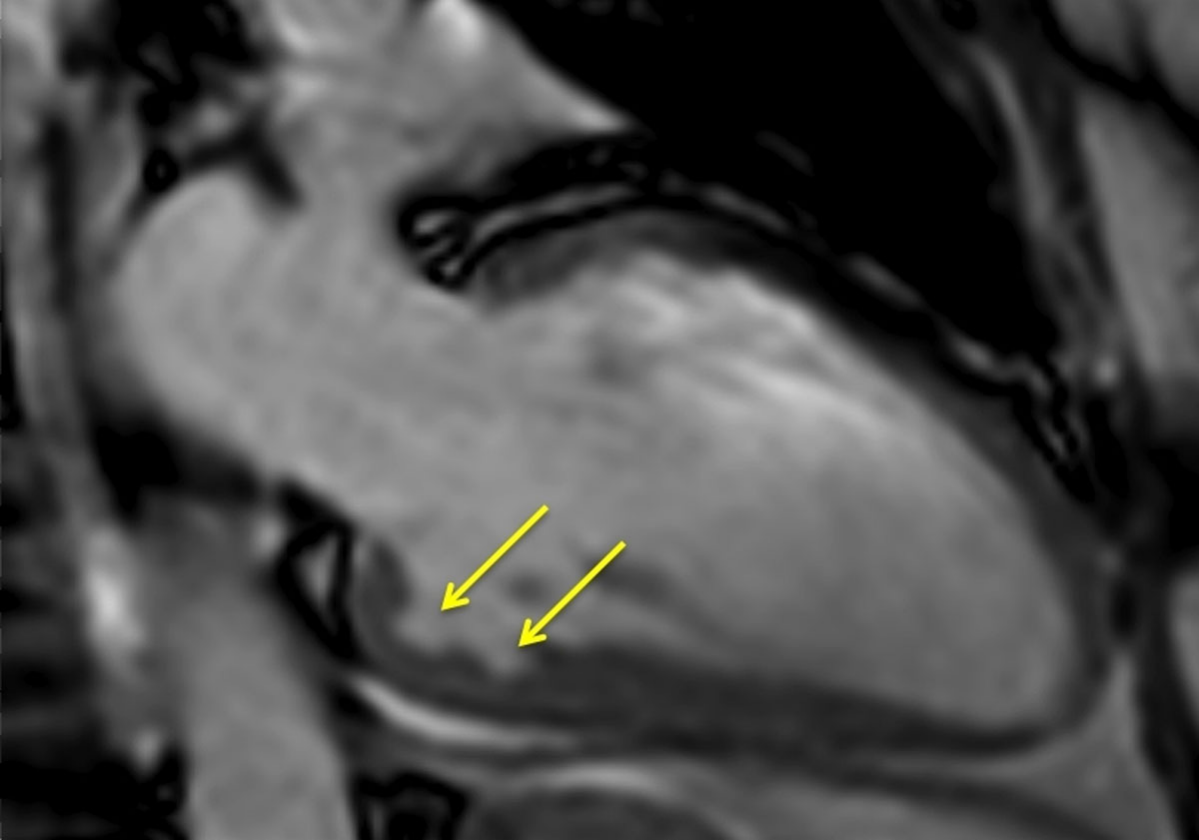
Multiple myocardial crypts in basal inferior segment.

**Figure 2:**
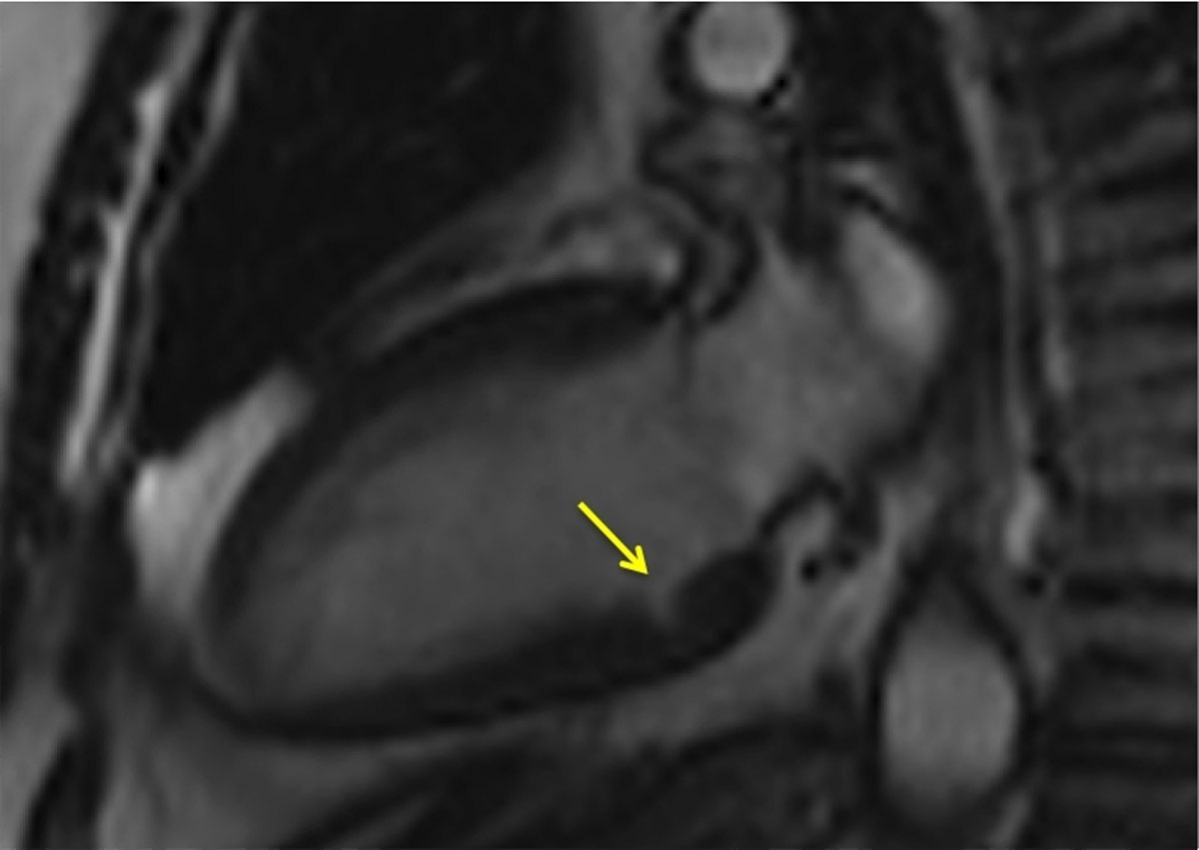
SIngle myocardial crypt in basal inferior segment.

